# Effects of Integrated Workplace Violence Management Intervention on Occupational Coping Self-Efficacy, Goal Commitment, Attitudes, and Confidence in Emergency Department Nurses: A Cluster-Randomized Controlled Trial

**DOI:** 10.3390/ijerph19052835

**Published:** 2022-02-28

**Authors:** Yang-Chin Chang, Mei-Chi Hsu, Wen-Chen Ouyang

**Affiliations:** 1 Department of Nursing, I-Shou University, Kaohsiung City 82445, Taiwan; ed100098@edah.org.tw (Y.-C.C.); hsu6889@gmail.com (M.-C.H.); 2 Department of Geriatric Psychiatry, Jianan Psychiatric Center, Ministry of Health and Welfare, Tainan City 71742, Taiwan; 3 Department of Nursing, Shu-Zen Junior College of Medicine and Management, Kaohsiung City 82144, Taiwan; 4 Department of Psychiatry, College of Medicine, Kaohsiung Medical University, Kaohsiung City 80708, Taiwan

**Keywords:** workplace violence, patient and visitor violence, emergency department, nurse, violence management, occupational coping self-efficacy, goal commitment, attitudes, confidence, randomized controlled trial

## Abstract

Patient and visitor violence (PVV), the most prevalent source of workplace violence, is largely ignored, underreported, and a persistent problem in emergency departments. It is associated with physical injuries, psychological distress, and occupational stress in nurses. A randomized controlled trial was conducted in Taiwan from January to December 2020. This study aimed to test the efficacy of an integrated Workplace Violence Prevention and Management Training Program on PVV in 75 emergency department (ED) nurses from a hospital. Cluster sampling was used because the policy of subdivision strategy was enforced during the COVID-19 pandemic. ED nurses received either the intervention or 1-hour in-service class. Data were collected from questionnaires. Data were analyzed mainly by the repeated measure analysis of variance and generalized estimating equations. The intervention had positive effects on developing stronger goal commitment, improving occupational coping self-efficacy, increasing confidence in ability to deal with violent situations, and modifying attitudes toward the causes and management of PVV in ED nurses (*p* < 0.05). The marginal *R*^2^ of the generalized estimating equation model for goal commitment, occupational coping self-efficacy, confidence, attitudes toward aggression in ED and aggressive behavior variables was high as 0.54 (*p* < 0.001), 0.45 (*p* < 0.001), 0.58 (*p* < 0.001), 0.29 (*p* < 0.05), and 0.72 (*p* < 0.001), respectively. These study models could effectively predict changes in the mean values. The benefit was driven by the effect of the intervention in ED nurses. Thus, the intervention, when applied in conjunction with routine in-service class, could exert synergistic improvements on outcomes measured in nurses.

## 1. Introduction

Workplace violence (WPV) in healthcare is persistent and often ignored [1,2]. Many people, when they experience medical events that pose an immediate risk to health or life, usually start with emergency medical services. Emergency departments (EDs) provide all aspects of urgent and critical pre-hospital medical care to patients. The recent coronavirus disease 2019 (COVID-19) pandemic has had significant impacts on both patients and health professionals [3]. The COVID-19 crisis further emphasizes the role of EDs in screening and management of patients with potential COVID-19 infection in both the short- and long-terms. Currently, reports on the prevalence of WPV in clinical medical practice vary significantly: 15–89.58% of workers were victims of verbal aggression/abuse; 18.22–56.0% of workers were victims of physical aggression, and 4.7–19.7% were victims of sexual harassment [4,5,6,7,8,9]. All these findings present an enormous risk of WPV for health providers.

There is a large body of evidence indicating that nurses are particularly at risk from violence exposure [1,3,7,9,10]. In EDs, 77% of staff reported experience of WPV [11]. Currently, COVID-19 which exacerbates WPV brought unique challenges for ED health professionals. A recent report has shown that nurses who provided care for patients with COVID-19 experienced higher rates of physical violence (adjusted odds ratio [aOR] = 2.18) and verbal abuse (aOR = 2.10) than who did not [3]. About 10% of them indicated that reporting the incident was even more difficult during the pandemic.

It has recently been reported that a series of violent injuries to ED health professionals in Taiwan have attracted a great deal of public attention. Similarly, WPV in healthcare has become widespread in Taiwan [12,13]. Over a 12-month period, 49.6% of nurses (13,392/26,979) reported at least one episode of any form of violence, 19.1% of nurses reported physical violence and 46.3% of them reported non-physical violence [12]. The prevalence of experiencing any violence varied widely across different healthcare settings; the highest (55.5%) was found in the emergency room or the intensive care unit [13].

As mentioned above, the definitions and descriptions of the sources and types of WPV varied. Experts and University of Iowa Injury Prevention Research Center have classified the workplace violence into four types based on the relationship between the perpetrator and the workplace itself [14,15,16]. The most common one is type II (patient and visitor violence, PVV), in which the perpetrators of violent incidents are patients and their visitors (relatives or friends) who become violent. Approximately 11.43–93% of respondents reported any form of violence, 22–90% reported verbal aggression, 2–32% reported physical aggression, and 12–64% of respondents experienced threat of violence in the previous 6, 12, or 24 [17,18,19,20,21,22].

To date, under-reporting of WPV or PVV remains an apparent problem. It results in ineffective resources and dilatory support for staff, which, in turn, may contribute to a higher incidence of burnout [1]. Of those identified as WPV victims, 65.6% did not formally report the episodes of violence through the hospital incident reporting (or other) systems and 86.1% did not report incidents of verbal abuse [23]. WPV can compromise the organizational functions due to decreased work performance and job satisfaction, increased job stress and turnover intention, impaired ability to handle workload, and burnout; it also has detrimental effects on recruitment, and work morale for nurses after being victimized [9,12,23,24,25,26,27]. These adverse impacts on nurses caused by WPV can translate into a decrease in the level of quality care, goal commitment, self-efficacy and nurses’ productivity, and an increase in the potential for medical errors or adverse events [1,12,23,28,29,30].

The lack of intervention studies is problematic. To date, most of studies on WPV and PVV in healthcare settings are mainly exploratory and descriptive in nature, focusing on correlational, retrospective, survey-based, or predictive designs. The majority of the literature reported the prevalence, sources, and types of WPV, and recruited a mixed professional groups or settings, and identified various and interrelated determinants, risks, contributory factors, and prevalence across various hospital settings in relation to WPV or other specific types of WPV. There were also studies examining whether implementation of workplace violence prevention interventions in EDs could reduce violent incidents or increase the knowledge or skills of ED staff. In spite of large variations in study design, study population, settings, components of interventions, and outcome measures, several positive research results were found. Most of these studies demonstrated positive effects on healthcare workers’ ability to deal with and assess violent situations [31,32,33,34], confidence to identify and respond to WPV [31,33,34,35,36], knowledge attainment [36], and coping strategies [31,33,34]. Results have also shown a decrease in financial impact of WPV [37], an increase in reporting assaults [38], and a reduction in the incidence of physical assaults and threats [39] and nurses’ turnover [40].

In light of the scant evidence evaluating the efficacy of WPV prevention and minimization strategies, development of training programs to effectively manage WPV in healthcare settings remains a challenge. We have also found very few studies focusing on the prevention and management of PVV in Eds, particularly relating to nurses. Further research on PVV, the impact of patient violence and nurses’ occupational outcomes are also needed.

### 1.1. Aims of the Study

Given heterogeneity of patients’ health care needs and the elevated stress because of the emergent conditions in EDs compared to wards, EDs are largely different from ward settings. These may impact the types of violence experienced, and the way to effectively deal with it.

A randomized controlled trial was carried out to evaluate in a more comprehensive way, the effects of a novel integrated Workplace Violence Prevention and Management Training Program (WPV-PMTP) on violence management of PVV. The trial has also examined the effect of this intervention on goal commitment, attitudes toward and confidence in managing workplace violence, and occupational coping self-efficacy in ED nurses. The research hypothesis was that WPV-PMTP, when applied in conjunction with routine in-service class, could exert synergistic effects on improvements in outcomes measured on ED nurses.

### 1.2. Theoretical Framework

The WPV-PMTP intervention is a multifaceted, multidisciplinary approach based on a theoretical model of Kraiger et al.’s [41] learning concept. Kraiger et al. [41] developed a systematic framework of learning outcomes and classified learning during training into three types of outcomes: cognitive, skill-based, and affective. Each type of outcome included particular foci of measurement. This conceptual framework has strengthened the development of the WPV-PMTP which offered structures of possible measures that later proved to be helpful and appropriate for this particular violence management training program. Accordingly, the study evaluation was structured around the measurement of nurses’ attitudes, self-efficacy, goal setting and perceived confidence.

Nearly 90% of workers reported that WPV had affected their work behaviors [42]. Thus, the occupational consequences of WPV warrant special attention from health professionals per se, managers, and healthcare organizations. Occupational coping self-efficacy is defined as self-appraisals of capabilities to deal with workplace specific stressful events, and to cope with workplace demands [43,44,45]. Several occupational stress studies have examined the job self-efficacy construct [43,44,46,47]. It includes two major issues. First, it focuses on personal beliefs about their ability to manage situational stress and challenges. Second, it closely relates to the coping abilities associated with the specific workplace stressors, such as WPV. Therefore, the focuses of occupational coping self-efficacy can be the ability to encounter the specific stressors in the job. It is somewhat different from more general job-related self-efficacy. More specifically, the concept of occupational coping self-efficacy for nurses includes a self-efficacy dimension concerning the belief in their capacities to deal with interpersonal relationship and conflicts at the workplace [44].

Nurses’ attitude toward violence is a critical element for effective management of WPV [31,48,49,50,51,52,53]. Since it possesses an important impact on their career, a right attitude can help them prevent, and manage the violent events and improve the quality of nursing care. In addition, WPV may not only cause negative health consequences, but also lead to loss of self-confidence and self-esteem among nurses [29,54]. A previous study investigated nurse’ perceptions of the incidence and nature of PVV has shown that 36% of victims of patient violence experienced emotional distress, 34% disbelief, 23% powerlessness, and 20% impaired job confidence [55].

## 2. Materials and Methods

### 2.1. Study Design

This study, conducted in 2020, was a cluster-randomized, pre- and post-test, controlled trial using parallel-groups of emergency department nurses in Taiwan. Different perpetrators were treated with different interventions through a range of strategies. Among various types of WPV, PVV is the most frequent form of violence [7,11,22,56]. In this study, intervention trial was focused on PVV. However, workplace bullying and/or inter-professional conflict were not included in the content of the WPV-PMTP in this study, as workplace issues were beyond the scope of PVV.

In the present study, the intervention group received a 12-session WPV plus standard 1-hour in-service class about hospital safety policies and procedures organized by the hospital. The control group received the standard 1-hour in-service class only, which maintained their routine training class at the hospital. A video conference was used because of the COVID-19 pandemic and subdivision strategy (team-based segregation in ED).

### 2.2. Setting, Participants, and Recruitment

The participating hospital which has more than 1200 beds, is one of the designated hospitals of COVID-19 in Taiwan. The emergency department has a number of functional areas that plays important parts in patient care. During the COVID-19 pandemic, the participating hospital has drawn up plans in preventing the spread of an outbreak by keeping groups of patients and health professionals apart. To avoid nosocomial infections and maintain the capacity of ED services, strategies, such as the subdivision strategy, modified isolation, and triage measures, etc., were applied to all health professionals. In particular, full-time nurses who provide care in each designated area of the emergency department were included. They were approached at a regular staff meeting or off-duty hour, and invited to participate in this study. The researchers managed the site recruitment process.

### 2.3. Sample Size and Estimated Study Power

To maximize statistical power, sample size estimation was calculated with a medium effect size of 0.25 for the outcomes, an alpha set at 0.05, and a power of 0.80, and result showed a requirement of 40 nurses for each group [57,58]. In this study, 39 and 36 participants were recruited and allocated randomly into the intervention and control groups, respectively.

### 2.4. Randomization

Cluster sampling was used because the policy of subdivision strategy was applied to health professionals during the COVID-19 pandemic. In this study, ED nurses were divided into groups (clusters) by the hospital, and formed the clusters for this study. Nurses in each cluster were randomly allocated into either intervention or control group. Nurses in each cluster could only fixed in one designated functional area. During the study, nurses were not expected to be transferred to another unit of the emergency department. Therefore, the risk of study contamination was minimal. However, blinding for this cluster-randomized controlled intervention trial was not possible because nurses who completed the baseline assessments and questionnaires were also personally involved in the study.

### 2.5. Intervention

This program (WPV-PMTP) enables staff to acquire data capacities in assessment, prevention and management of WPV. This 12-session course had 12 components. Each session lasted at least 1 h. The core contents of the intervention were centered first on the improvement of nurses’ awareness of WPV, such as identification of patients/visitors at high-risk of violent behavior, the motivations of perpetrators, the causal factors for PVV (internal, external, and interactional causes), possible triggers of violence, and cues to impending aggression. Secondly, the intervention focused on interaction, management, prevention, and post-incident action, such as danger assessment, communication skills for a potentially threatening situation, problem solving, conflict management, and anger management. Finally, the intervention facilitated nurses to be competent in developing assertiveness techniques, engaging in more complicated interactions, and proactive violence management. A regular team-debriefing and feedback was implemented. A checklist was also provided to nurses for helping them remember key components in the intervention. The researchers specified what nurses could achieve after the completion of intervention, such as the complement or correction of deficiencies in capacity and performance.

The video conference was highly Interactive in this study. Lecturers, including the researchers, a psychiatrist, and a social worker, used a variety of teaching methods, including proactive questioning, role-plays, scenario examples based on actual WPV, in various communication exercises, discussions, and debriefing.

This program (WPV-PMTP) was developed by the research team. The research team comprised a nurse supervisor with about 20-years of experience in hospitals, a nurse educator with more than 20-years of experience in university and hospital psychiatric wards, and a psychiatrist with about 30-years of experience from the psychiatric center. The research team members were familiar with and well trained on violence prevention and management strategy, and delivered integrity and credibility to all aspects of clinical trial conduct.

### 2.6. Outcome Measures

This study incorporated several assessments. Selection of outcome measures followed the conceptual framework and the purpose of the study. Questionnaires included demographics, goal commitment, occupational coping self-efficacy, attitudes toward aggression in the emergency room, and confidence in managing aggressive behavior.

Goal Commitment Scale: The 5-item goal commitment scale [59] was used to measure the accomplishment and commitment of goal tasks. Participants indicated their agreement to each item on a 5-point Likert scale varied from “I agree a lot” to “I disagree a lot”. A higher score refers to a higher level of goal commitment. Cronbach’s α was 0.833.

Occupational Coping Self-Efficacy Questionnaire for Nurses (OCSE-N): The 9-item OCSE-N was used to assess two distinct occupational coping self-efficacy beliefs: occupational burden and relational difficulties at the workplace [44]. The OCSE-N used a 5-point Likert scale ranging from 1 (not at all easy to cope with) to 5 (extremely easy to cope with). The total scores were summed with a higher score indicating stronger beliefs regarding how confident nurses feel they can cope with occupational stressful situations and actively address challenges. The Cronbach’s α of 0.930 was high [47].

Attitudes Toward Aggression in Emergency Room: The 16-item Attitudes Toward Aggression in Emergency Room was used to identify ED nurses’ attitudes and thoughts about factors related to violence in ED [60]. Cronbach’s α was 0.83. Score of each item ranged from 1 (“strongly disagree”) to 4 (“strongly agree”). The total scores were summed up with a higher total score indicating a more positive attitude.

Confidence in Managing Aggressive Behavior: This 8-item survey questionnaire was used to measure nurses’ confidence levels in managing aggressive behavior [60]. Each item was rated on a 4-point scale, ranged from 1 (not) to 4 (extremely). The total scores were summed up with a higher score indicating a greater confidence to the management of PVV. Cronbach’s α was 0.83.

Attitudes Toward Aggressive Behavior Questionnaire (ATABQ): The ATABQ was used to measure nurses’ attitudes on prevention and management of patient aggressiveness [61]. The questionnaire includes 12 items covering 5 themes: prediction, patient motivation and responsibility for aggression, staff anxiety and fear of assault, the need for skilled intervention to prevent and manage aggression, and staff confidence. Each item was rated on a 5-point Likert scale from strongly disagree to strongly agree. The scores were then summed up. A higher total score indicated a more positive attitude. A high test–retest stability (r = 0.972) has been established.

### 2.7. Ethical Considerations

The study protocol for this trial was approved by the Institutional Review Board (IRB) of the participating hospital (registered No. EMRP42108N). Prior to the study, written informed consent in conformity with ethical approval was secured from all participants. Throughout the study period, researchers have ensured the rights, health, and well-being of participants, and all study procedures were conducted in accordance with the ethical principles of the 1975 Declaration of Helsinki.

### 2.8. Data Analysis

Statistical analyses were performed using software, SPSS version 22.0, for Windows (IBM). Descriptive statistics were used for comparative analyses of the samples and groups. Demographic characteristics were presented as numbers along with percentages. Mean and standard deviation (SD) were calculated for continuous variables. The chi-square test (χ^2^) was applied for categorical data. The independent *t*-test was used to examine group differences. A repeated measure analysis of variance (ANOVA) was used to compare the mean differences between the intervention and control groups in order to identify any changes in study outcomes. Data were finally analyzed using generalized estimating equations (GEE) because of the possible correlation between outcomes from repeated measures at baseline and post-test. The GEE analyses were also used to estimate working correlation parameters for total sample, and detect differences between the intervention and control groups. A significant group x time interaction-effect indicates that the intervention group significantly differs from the control group in a specific outcome. The GEE models consider baseline data and the changes between outcomes over time in different groups. The effect of the WPV-PMTP was estimated after adjusting for the following covariates: age, experience of PVV, and work tenure as a nurse in ED. We also compared the marginal R^2^ statistic for models to assess the contribution of selected variables. The conclusions about the effects of the intervention were based on GEE from baseline to post-test. The level of significance was set at *p* < 0.05.

## 3. Results

Demographic characteristics of ED nurses were shown in Table 1. Comparisons of characteristics, such as marriage status, religion, job title, professional level (rank), and experience of PVV in nurses showed no significant differences between two groups (*p* > 0.05). However, there were significant differences (*p* < 0.05) in age, education, and years of experience (work tenure), and a borderline significant difference in gender between groups (*p* = 0.050).

Table 2 shows that the goal commitment scores in the intervention group as compared to those in the control group, were significantly improved at post-test (*p* < 0.001). A statistical trend in developing a strong commitment to achieve a goal, and stop abandoning the goal was also observed in the intervention group (*p* < 0.001) compared to the control group. A significant improvement in occupational coping self-efficacy was also found in the intervention group at post-test (*p* < 0.001). Nurses in the intervention group reported that they coped more easily with occupational stressful violent situations. They were also more confidence in their ability to manage a patient/relative’s violent behavior (*p* < 0.001). These significant improvements included communicating properly with people involving violent event, responding to a patient/relative who have threat or manipulation behavior, managing with violent situations, and finally working as a team member in responding to violent situations (*p* < 0.001).

Nurses in the intervention compared to control group have also shown a significant improvement in attitudes toward issues relating to violence in ED (*p* = 0.020), and an increase of the feeling of being supported from management. In this study, WPV-PMTP demonstrated significant improvements in attitude toward violent behavior and explanation of violence (*p* < 0.001). This was reflected by significant increases in overall global scores (*p* < 0.001) and 4 subscales after the completion of the WPV-PMTP. Furthermore, nurses also showed a strong belief in the need for capacity to prevent and manage violent behavior (*p* < 0.001). Nurses in the intervention group reported that they were less nervous, and were able to think straight when a patient/relative becomes aggressive (*p* < 0.001). They were also more likely to agree that doing the wrong things would make a bad situation worse (*p* = 0.021).

The repeated measure ANOVA analyses showed statistically significant time-by-group interaction effects on goal commitment (*p* < 0.001), occupational coping self-efficacy (*p* < 0.001), confidence (*p* < 0.001), attitudes toward aggressive behavior (*p* < 0.001), and 5 subscales of ATABQ (prediction, *p* < 0.001; patient motivation and responsibility for aggression, *p* < 0.001; staff anxiety and fear of assault, *p* < 0.001; the need for skilled intervention to prevent and manage aggression, *p* = 0.001; staff confidence, *p* = 0.030) (Table 3). As time increased, these differences became even more apparent. Thus, the intervention group, as compared to the control group, show significant improvements over time. However, there were no significant effects on attitudes toward aggression in ED (*p* = 0.061).

The effectiveness of WPV-PMTP on study outcomes based on GEE analyses are shown in Table 4. For goal commitment, the significant group x time interaction revealed that nurses in the intervention group as compared to the control group had a greater improvement in goal commitment after completion of WPV-PMTP (*Β* = 4.02, *p* < 0.001, 95% CI = 3.13–4.91, Marginal *R*^2^ = 0.54). Moreover, nurses in the intervention group compared to the control group had a significant increase in occupational coping self-efficacy (*Β* = 4.83, *p* < 0.001, 95% CI = 2.76–6.91, Marginal *R*^2^ = 0.45). Nurses in the intervention group also had a significant increase in confidence in managing violence (*Β* = 4.58, *p* < 0.001, 95% CI = 2.71–6.45, Marginal *R*^2^ = 0.58) and attitudes toward aggressive behavior (*Β* = 9.15, *p* < 0.001, 95% CI = 7.39–10.91, Marginal *R*^2^ = 0.72). However, attitudes toward aggression in ED compared to the control group showed only a borderline of statistical significance (*Β* = 1.67, *p* = 0.050, 95% CI = −1 × 10^−3^–3.33, Marginal *R*^2^ = 0.29).

## 4. Discussion

A proper preventive orientation that focus on adequate indicators and outcomes, is the key to a successful intervention and may extend to active goal outcome. In the present multifaceted intervention, which involved professional training in workplace violence prevention and management, was designed to improve occupational coping self-efficacy, confidence, goal commitment and attitudinal changes. The statistically significant improvements in the outcomes were observed among nurses receiving WPV-PMTP but not those with routine in-service class only. The positive outcomes indicate that WPV-PMTP is well received and making a clinically meaningful contribution in this area.

We found that WPV-PMTP, compared to the control, showed significant improvements in some of the cognitive-affective domains, such as attitudes and confidence, goal commitment, and occupational coping self-efficacy beliefs. The positive effects on these outcomes were consistent with findings from other studies conducted in EDs [32,39,60,62], hospital settings [31,63,64,65,66] and community [67]. Furthermore, nurses in the WPV-PMTP group were also confident in their capacities to assess violent situations, and to manage violent situations increased. These positive results are also consistent with previous studies [31,32,33,34]. Thus, our findings provided a useful and significant information on evaluation of the effect of the intervention.

As demonstrated in this study, most nurses reported a lower level of occupational coping self-efficacy at the baseline. They reported a marked amount of distress in occupational stressful situations with which they found it more difficult to cope, WPV-PMTP yielded positive effects in occupational coping self-efficacy to deal with WPV, and coping strategies which they felt they became easier to cope with occupational stressful situations. It is evident from this study that WPV-PMTP helped to improve these predicaments. The effect seemed to occur immediately after the WPV-PMTP training.

There are several possible explanations for the positive outcomes. First, the WPV-PMTP has an effect on the improvement of occupational coping self-efficacy. Indeed, studies have found support for the notion that the impact of job-related psychological distress may be associated with low coping self-efficacy [43,47,68]. Nurses’ perceived occupational coping self-efficacy may not only be a capacity, but also a specific functioning. Research has shown that nurses, who believe themselves incapable of overcoming threatening workplace events, generally have decreased distress and higher occupational self-efficacy beliefs [69,70].

Second, occupational coping self-efficacy can buffer the impact of WPV on work-related outcomes, thereby diminishing negative consequences on nurses exposed to WPV [43]. In other words, nurses with high levels of occupational coping self-efficacy show less psychological distress and emotional exhaustion related to exposure to WPV [43,45].

Third, occupational coping self-efficacy may present both a direct and an indirect effect on emotional well-being [44]. A study further indicated that occupational coping self-efficacy among nurses could explain 1–4% of the additional variance in one of the indicators of wellbeing/distress/emotional exhaustion [43]. Thus, positive task-oriented coping strategies which can reduce distress levels may be promoted.

Finally, WPV-PMTP may help nurses enhancing their coping strategies to identify and manage characteristics and risk of PVV. As such, it is important to provide ED nurses with effective workplace violence prevention interventions to improve related techniques for managing WPV.

This study demonstrated the importance of considering task specific self-efficacy in occupational research, and suggested that the assessment of self-efficacy beliefs should be tailored to the particular domain related to WPV prevention.

Results in this study show that nurses who received WPV-PMTP having higher scores of confidence in managing WPV in their post assessment. This finding is similar to other studies which found improved confidence when dealing with violence after a violence prevention training program [21,49,52,53,65,71]. It is evident that training increases nurse’s confidence in managing an escalating patient. WPV-TPMP served as a tool that enables nurses in recognizing and responding to WPV with suitable strategies. The WPV-TPMP also involved in participatory experience, facilitating nurses to confidently enhance their levels of awareness and problem-solving strategies during the training, and apply these strategies to manage the WPV situations. Our findings are compatible to those in the above-mentioned studies, indicating that WPV-TPMP is applicable to workplace violence in nursing.

Goal commitment is an important component in goal management and relationship between goals and real nursing performance. It also plays an influential role on the process of attempting to achieve a desired future outcome and decision-making [72,73,74]. Results in the present study show the effectiveness of the WPV-PMTP on violence management, and the improvements in goal commitment. The participation of WPV-TPMP has heightened the nurses’ commitment to prevent and manage violence. Whenever an individual commits to a goal, his/her performance will always be higher. Goal commitment to do something, responsibility, or enthusiasm may carry a promise for the individual to follow through [75]. We have also found in this study that goal commitment has a significant influence on completion of the training program. This implicates for nursing practice that building commitment-boosting strategies in order to boost their commitment for WPV prevention, will increase their odds to successfully curb and manage WPV.

Self-efficacy, or self-confidence are two main factors that could influence goal commitment [72,73,76]. Self-efficacy and goal commitment are closely linked as self-efficacy enhances an individual’s commitment to difficult goals and actions [72,75,76]. Based on the findings in the present study, goal commitment, self-efficacy, and confidence are important components of actions in WPV management. They reflect the importance of goal commitment for WPV related tasks and provide a better understanding of the mechanisms of the linkage of goal commitment, self-efficacy and confidence.

Our result shows the attitudes toward WPV yielded a *p*-value of 0.05 which represents a borderline of statistical significance. At the baseline, two groups of ED nurses were same on attitudes toward aggression. However, the differences (although borderline significance) in attitude scores would, nevertheless, infer that WPV-PMTP is effective. The more positive attitudes would lead to better implement in prevention and mitigation strategies, and decrease the overall risk to WPV.

Before considering the factors that influence attitudes toward violence in EDs, it is important to consider the broader literature on the under-reporting issue. A recent study in Taiwan found that only a very small proportion of the victims (4.9–12%) completed the incident report. The main reason for not reporting violent incidents in wards they encountered was their belief that such a report was useless or unimportant [24]. Alyaemni and Alhudaithi [77] have found that most nurses did not want to report incidents (47.2%), considering it useless (nothing would be done) or unimportant (15%). The under-reporting indirectly reflected nurses’ attitudes towards the WPV that they considered the reporting was of little value since nothing would be done or they were dissatisfied with how the incident was handled. Although such attitudes toward WPV have improved in recent years [48], their attitudes remained negative and continued in not reporting WPV or taking the necessary actions. Unfortunately, the lack of reporting leads to nurses being unable to obtain timely support.

However, the present study revealed that intervention has significantly increased the mean scores of a single item within the attitude scale regarding communication styles in management of WPV. WPV-TPMP has led to a meaningful improvement of attitudes in communication. It is possible that WPV-TPMP changes the perception and the recognition of WPV, and, therefore, mitigates the risk of PVV. To accurately confirm the effects of attitude changes, it is recommended that future research needs a larger sample size and longer follow-up times.

Our study demonstrates that WPV-PMTP could ameliorate nurses’ attitudes toward prediction of PVV, and increase nurses’ attitudes to become more positive. This finding is consistent with a previous study by Wong et al. [78]. Prediction through proper appraisal of PVV, particularly based on clinical features is the emphasis of our intervention. The nurses’ attitudes toward the possibility of prediction of WPV was becoming positive at post-intervention. Nurses responded that violence can be preventable through which to observe and understand the behavior of patients/visitors.

The effectiveness of the WPV-PMTP may also relate to improvements in nurses’ attitudes toward patient motivation and responsibility for aggression. The WPV-PMTP in the present study had heightened nurses’ levels of awareness of the patient’s factors that contribute to WPV. Nurses reported more positive attitudes toward patients who become aggressive or violent when they feel vulnerable, helpless, and afraid as measured by the ATABQ. Cognitive impairment, confusion, delirium, dementia, and alcohol and illegal drug intoxication or withdrawal are often detected in violent patients [1,20,21,79,80]. These illnesses and cognitive status limit patients’ ability to communicate effectively and understand the situation as the disease or discomfort progresses and its context during stay in ED.

In the present study, WPV-PMTP has substantial improved the perceptions of being nervous when a patient becomes increasingly aggressive. Nurses in the intervention group reported a reduction in the fear of assault. However, Spector et al. [81] demonstrated that their training did not influence experienced stress.

In this study, the improvement of anxiety and fear may also reflect a positive attitude change and strengthened confidence and self-efficacy. In other words, nurses who received WPV-PMTP, in contrast to those who do not, showed less anxious and more satisfied with information and capacities obtained and fewer adjustment problems. These responses are expected to contribute to the reduction in PVV incidences since nurses with the anxiety trait is a significant contributor to type II WPV in clinical settings.

Results in this study have also demonstrated the significant effects of WPV-PMTP on nurses’ positive attitudes towards the needs of training for specific skills to prevent and manage violence. The findings are similar to those reported elsewhere [31,32,33,34]. Awareness and preparation are keys to managing WPV in EDs and maintaining a safe and reliable working environment. The provision of sufficient education and training programs for ED nurses is critical to increase their awareness of potential violence risks and respond effectively when situations escalate.

Overall, the findings of the present study showed that WPV-PMTP significantly improved mean scores of goal commitment, occupational coping self-efficacy, confidence, and attitudes of the intervention group as compared with the control group. Therefore, WPV-PMTP is a promising approach for prevention and management of WPV.

There are strengths in the present study. The specificity of the WPV-PMTP, which focused exclusively on PVV, is a major strength of this study since PVV is the most common style of WPV in ED. Another strength is that it measured several elements related to WPV among ED nurses. This provides a further understanding related to mechanisms of the intervention. However, a longer follow-up study is suggested, as the effect of intervention seems to increase over time. The implementation of follow-up plans to prevent future occurrences of PVV is also recommended.

### Study Limitations

Our study had limitations, such as that the WPV-PMTP focused mainly on PVV, and did not fully investigate other types of WPV. This decreases the generalizability of the study. Since WPV-PMTP is a multifaceted intervention, it is not possible to distinguish which of the components were primarily responsible for the observed outcomes. Moreover, education has been often used as a covariate in the analysis of clinical trials. More research effort is required to include education as covariates in analyzing intervention effects in trials. Finally, because of COVID-19 pandemic the implementation of subdivision strategy which keeps nurses apart in different areas may impact on the cluster randomization of this study, resulting in significant differences in some demographic characteristics at baseline. The researcher team may not have had considerable flexibility in selecting the unit of randomization. Thus, the randomization remains a limitation of this study.

## 5. Conclusions

The WPV-PMTP intervention had positive effects in developing stronger goal commitment, improving occupational coping self-efficacy, increasing confidence in ability to deal with violent situations, and modifying attitudes toward the causes for and management of PVV in ED nurses. These positive results have largely been achieved by raising the awareness of ED nurses to the nature of PVV, developing their capacities in managing violent situations, and improving their attitudes toward potentially violent incidents and perpetrators. This study confirms that prevention and management of PVV is important since PVV is particularly and substantively associated with negative consequences. It also provides a foundation for further research in this field. Future studies should consider organizational interventions for a better assessment of the effects of prevention and management of PVV in ED.

## Figures and Tables

**Table 1:** Baseline characteristics of participants randomized to the training intervention and control conditions.

	Intervention Group (*n* = 39)	Control Group (*n* = 36)	*p*
	*n*/%	*n*/%	
Gender			0.050
Male	1 (2.56)	6 (16.67)	
Female	38 (97.44)	30 (83.33)	
Age: M (SD) (years)	33.74 ± 6.48	29.14 ± 4.86	0.001
21~30	20 (51.28)	30 (83.33)	0.007
31~40	10 (25.64)	5 (13.89)	
41~50	9 (23.08)	1 (2.78)	
Education			0.030
Junior college	18 (46.15)	8 (22.22)	
≥University	21 (53.85)	28 (77.78)	
Marriage status			0.307
Single	25 (64.10)	27 (75.00)	
Married	14 (35.90)	9 (25.00)	
Religion			0.689
None	16 (41.03)	13 (36.11)	
Buddhism/Taoism/others	21 (53.85)	19 (52.78)	
Christian/Catholics	2 (5.13)	4 (11.11)	
Job title			1.000
Registered nurse	37 (94.87)	34 (94.44)	
Nurse leader	2 (5.13)	2 (5.56)	
Professional level (rank)			0.059
N0	23 (58.97)	16 (44.44)	
N1	4 (10.26)	12 (33.33)	
N2	11 (28.21)	8 (22.22)	
N3	1 (2.56)	0 (0.00)	
Working experience in ED (years)	7.24 ± 4.86	4.29 ± 3.32	0.003
Working experience in ED of the participating hospital (years)	6.53 ± 4.94	4.10 ± 2.91	0.011
Have experienced patient/visitor violence in the past year			0.087
Yes	10 (25.64)	16 (44.44)	
No	29 (74.36)	20 (55.56)	
Have experienced patient/visitor verbal violence in the past year			0.087
Yes	10 (25.64)	16 (44.44)	
No	29 (74.36)	20 (55.56)	
Have experienced patient/visitor physical violence in the past year			1.000
Yes	4 (10.26)	4 (11.11)	
No	35 (89.74)	32 (88.89)	

**Table 2:** Performance on outcomes of training efficacy.

	Intervention Group (*n* = 39)		Control Group (*n* = 36)		Test Statistics		Mean Differences	
	Baseline	Post-Test	Baseline	Post-Test	Baseline	Post-Test	Intervention Group	Control Group
	M ± SD	M ± SD	M ± SD	M ± SD			M ± SD	M ± SD
Goal commitment	13.36 ± 1.90	17.46 ± 1.29	12.92 ± 2.06	13.00 ± 2.04	0.337	<0.001	4.10 ± 2.04	0.08 ± 1.95
Occupational coping self-efficacy	21.9 ± 3.96	28.9 ± 3.27	20.4 ± 3.69	22.6 ± 4.11	0.089	<0.001	7.00 ± 4.81	2.17 ± 4.49
Confidence	18.72 ± 3.32	27.41 ± 2.56	20.56 ± 3.89	24.67 ± 2.31	0.030	<0.001	8.69 ± 4.47	4.11 ± 3.92
Attitudes toward aggression in ED	46.26 ± 2.78	50.59 ± 4.20	45.94 ± 3.36	48.61 ± 2.89	0.662	0.020	4.33 ± 4.44	2.67 ± 2.93
Attitudes toward aggressive behavior	29.92 ± 2.79	40.77 ± 3.62	28.83 ± 3.27	30.53 ± 2.79	0.124	<0.001	10.85 ± 4.64	1.69 ± 3.16
Prediction	5.49 ± 1.43	7.77 ± 1.06	5.06 ± 1.26	4.97 ± 1.46	0.172	<0.001	2.28 ± 1.92	−0.08 ± 1.44
Patient motivation and responsibility for aggression	9.46 ± 1.68	12.67 ± 1.51	9.03 ± 2.10	9.14 ± 2.11	0.326	<0.001	3.21 ± 2.05	0.11 ± 1.86
Staff anxiety and fear of assault	5.49 ± 1.43	7.36 ± 1.04	5.36 ± 1.33	5.47 ± 1.38	0.695	<0.001	1.87 ± 1.61	0.11 ± 1.53
The need for skilled intervention to prevent and manage aggression	7.59 ± 1.60	9.72 ± 1.26	7.14 ± 1.40	7.83 ± 1.48	0.200	<0.001	2.13 ± 1.81	0.69 ± 1.79
Staff confidence	1.90 ± 0.72	3.26 ± 0.44	2.25 ± 0.60	3.11 ± 0.67	0.025	0.266	1.36 ± 0.99	0.86 ± 0.96

**Table 3:** Effects of the integrated Workplace Violence Prevention and Management Training Program (WPV-PMTP) on study outcomes.

	Group (Inter-Group) Effect ^a^		Time (Within) Effect ^b^		Interaction of Group and Time ^c^	
	F	*p*	F	*p*	F	*p*
Goal commitment	46.91	<0.001	82.47	<0.001	76.03	<0.001
Occupational coping self-efficacy	33.29	<0.001	72.45	<0.001	20.14	<0.001
Confidence	0.76	0.385	172.87	<0.001	22.13	<0.001
Attitudes toward aggression in ED	3.18	0.079	63.85	<0.001	3.62	0.061
Attitudes toward aggressive behavior	102.21	<0.001	184.20	<0.001	98.10	<0.001
Prediction	49.05	<0.001	31.07	<0.001	35.96	<0.001
Patient motivation and responsibility for aggression	29.41	<0.001	53.31	<0.001	46.40	<0.001
Staff anxiety and fear of assault	17.52	<0.001	29.87	<0.001	23.55	<0.001
The need for skilled intervention to prevent and manage aggression	20.19	<0.001	46.14	<0.001	11.90	0.001
Staff confidence	1.42	0.237	97.22	<0.001	4.89	0.030

Note: ^a^ Reference: control group; ^b^ Reference: baseline values; ^c^ Reference: baseline values of control group.

**Table 4:** Generalized estimating equation analyses of the outcome measures from baseline to post-test based on the Training Program.

	*β*	95% Confidence Interval	*p*	Marginal *R*^2^ (*R*^2^marg)
Goal commitment				0.54
Group (intervention vs. control)	0.66	(−0.18–1.49)	0.123	
Time	0.08	(−0.54–0.71)	0.795	
Age	−0.01	(−0.07–0.05)	0.770	
Work tenure as a nurse in ED	−0.04	(−0.14–0.06)	0.447	
Experience of PVV (yes vs. no)	0.32	(−0.47–1.12)	0.427	
Group × Time	4.02	(3.13–4.91)	<0.001	
Occupational coping self-efficacy				0.45
Group (intervention vs. control)	1.52	(−0.24–3.28)	0.090	
Time	2.17	(0.72–3.61)	0.003	
Age	−0.07	(−0.20–0.07)	0.337	
Work tenure as a nurse in ED	0.13	(−0.06–0.31)	0.173	
Experience of PVV (yes vs. no)	0.31	(−1.26–1.88)	0.700	
Group × Time	4.83	(2.76–6.91)	<0.001	
Confidence				0.58
Group (intervention vs. control)	−1.44	(−3.16–0.29)	0.103	
Time	4.11	(2.85–5.37)	<0.001	
Age	−0.02	(−0.13–0.08)	0.688	
Work tenure as a nurse in ED	−0.04	(−0.17–0.10)	0.600	
Experience of PVV (yes vs. no)	1.04	(−0.05–2.13)	0.061	
Group x Time	4.58	(2.71–6.45)	<0.001	
Attitudes toward aggression in ED				0.29
Group (intervention vs. control)	0.78	(−0.63–2.20)	0.279	
Time	2.67	(1.72–3.61)	<0.001	
Age	−0.04	(−0.16–0.09)	0.580	
Work tenure as a nurse in ED	4 × 10^−3^	(−0.20–0.21)	0.970	
Experience of PVV (yes vs. no)	1.69	(0.31–3.07)	0.017	
Group × Time	1.67	(−1 × 10^−3^–3.33)	0.050	
Attitudes toward aggressive behavior				0.72
Group (intervention vs. control)	0.74	(−0.50–1.98)	0.244	
Time	1.69	(0.68–2.71)	0.001	
Age	−0.03	(−0.13–0.07)	0.589	
Work tenure as a nurse in ED	0.14	(−0.01–0.29)	0.075	
Experience of PVV (yes vs. no)	−0.38	(−1.65–0.90)	0.565	
Group × Time	9.15	(7.39–10.91)	<0.001	

## Data Availability

The data presented in this study are available on request from the corresponding author. The data are not publicly available due to privacy and ethical restrictions.
